# A NADE nomogram to predict the probability of 6-month unfavorable outcome in Chinese patients with ischemic stroke

**DOI:** 10.1186/s12883-019-1464-6

**Published:** 2019-11-07

**Authors:** Chao Sun, Xiang Li, Baili Song, Xiangliang Chen, Linda Nyame, Yukai Liu, Dan Tang, Mako Ibrahim, Zheng Zhao, Chao Liu, Miao Yan, Xiding Pan, Jie Yang, Junshan Zhou, Jianjun Zou

**Affiliations:** 1 School of Basic Medicine and Clinical Pharmacy, China Pharmaceutical University, Nanjing, Jiangsu China; 2 Department of Clinical Pharmacology, Nanjing First Hospital, Nanjing Medical University, Nanjing, Jiangsu China; 3 Department of Neurology, Nanjing First Hospital, Nanjing Medical University, Nanjing, China; 4 Department of Pharmacy, the Second Xiangya Hospital, Central South University, Changsha, China; 5 Department of Neurology, the First Affiliated Hospital of Chengdu Medical College, Chengdu, China

**Keywords:** Stroke, Cerebral ischemia, Unfavorable outcome, Prediction, Nomogram

## Abstract

**Background:**

Early prediction of unfavorable outcome after ischemic stroke is of great significance to the clinical and therapeutic management. A nomogram is a better visual tool than earlier models and prognostic scores to predict clinical outcomes, which incorporates different factors to develop a graphic continuous scoring system and calculates accurately the risk probability of poor outcome entirely based on individual characteristics. However, to date, no nomogram models have been found to predict the probability of 6-month poor outcome after ischemic stroke. We aimed to develop and validate a nomogram for individualized prediction of the probability of 6-month unfavorable outcome in Chinese patients with ischemic stroke.

**Methods:**

Based on the retrospective stroke registry, a single-center study which included 499 patients from May, 2013 to May, 2018 was conducted in Nanjing First Hospital (China) for ischemic stroke within 12 h of symptoms onset. The main outcome measure was 6-month unfavorable outcome (mRS > 2). To generate the nomogram, **N**IHSS score on admission, **A**ge, previous **D**iabetes mellitus and cr**E**atinine (NADE) were integrated into the model. We assessed the discriminative performance by using the area under the curve (AUC) of receiver-operating characteristic (ROC) and calibration of risk prediction model by using the Hosmer–Lemeshow test.

**Results:**

A visual NADE nomogram was constructed that NIHSS score on admission (OR: 1.190, 95%CI: 1.125–1.258), age (OR: 1.068, 95%CI: 1.045–1.090), previous diabetes mellitus (OR: 1.995, 95%CI: 1.236–3.221) and creatinine (OR: 1.010, 95%CI: 1.002–1.018) were found to be significant predictors of 6-month unfavorable outcome after acute ischemic stroke in Chinese patients. The AUC–ROC of nomogram was 0.791. Calibration was good (*p* = 0.4982 for the Hosmer–Lemeshow test).

**Conclusion:**

The NADE is the first nomogram developed and validated in Chinese ischemic stroke patients to provide an individual, visual and precise prediction of the risk probability of 6-month unfavorable outcome.

## Background

Globally, stroke is now the second most common cause of death and a major cause of disability [1, 2], and despite a reduction in mortality and hospitalization owing to improved acute management and implementation of preventive measures, the rate of stroke is on the rise owing to aging population. Therefore, early prediction of unfavorable outcome after ischemic stroke is of great significance to provide a reasonable approach to the clinical and therapeutic management and also help the patients and their families in understanding the challenging consequences of cerebral ischemia.

Nomogram is a visual statistical instrument that incorporates different data to develop a continuous scoring system which reflects the individual and precise risk probability. The accurate continuous probability power of the score emphasizes the potential of nomogram score as an important part of modern medical decision-making and a useful risk stratification tool that has been applied in routine clinical practice including cancer and surgery [3–6].

To our knowledge, there are only three researches on nomograms for individualized prediction of poor outcome of ischemic stroke in Caucasians (Italian) [7–9]. The START nomogram is developed and validated for individualized prediction of 3-month unfavorable outcome in intravenous thrombolysis-treated stroke patients [7]. Another nomogram model was established in 344 patients who started oral anticoagulants 1–7 days after atrial fibrillation-related stroke onset to predict the probability of 3-month poor outcome [8]. They found that NIHSS score which reflects the severity of stroke is the strongest predictor of 3-month unfavorable outcome [7–9]. However, to date, no nomogram model has been established to predict the probability of 6-month (long-term) unfavorable outcome after ischemic stroke in Chinese patients. Furthermore, the correlation of NHISS score with the outcomes varies with the time passed from the onset of cerebral ischemia [10]. It is unclear how National Institute of Health stroke scale (NHISS) score and other risk factors affect the 6-month (long-term) unfavorable functional outcome.

The objectives of this study were to develop and validate a nomogram model based on the integration of different degrees of stroke severity and other risk factors for individualized prediction of the probability of 6-month (long-term) unfavorable functional outcome after ischemic stroke in Chinese patients, which can help direct individual treatment for ischemic stroke patients.

## Methods

### Study design, participants, and procedures

Based on the retrospective stroke registry, a single-center study was conducted in Nanjing First Hospital, Nanjing Medical University, China. The study protocol was approved by the hospital medical ethics committee in accordance with the Helsinki declaration. All of the consecutive patients were recorded in the stroke registry between May, 2013 and May, 2018 for ischemic stroke within 12 h of symptoms onset.

All patients managed with endovascular procedures were excluded from the study. Patients with signs of intracranial hemorrhage (ICH) on baseline brain computed tomography (CT) scan, age < 18 years, lack of 6-month modified Rankin Scale (mRS) score and NIHSS score on admission unknown were excluded from the analysis. On admission, all clinical, demographic and laboratory characteristics were collected, including: age, sex, baseline NIHSS score, creatinine, platelet count, total cholesterol, fasting blood glucose, interval from onset to hospital within 4.5 h, medical history such as diabetes mellitus, hypertension, valvular heart disease, cerebral hemorrhage and so on. The quality of laboratory data was validated throughout the study period by regular internal quality control procedures and participation to an External Quality Assessment scheme. Baseline NIHSS and 6-month mRS were assessed by trained physicians with telephone questionnaires or face-to-face interviews. Mortality, and any complications were recorded. The primary outcome was unfavorable functional outcome after ischemic stroke, as reflected by a mRS comprised between 3 and 6 (i.e., poor prognosis) at 6-month [11].

### Statistical analysis

Baseline characteristics were summarized with descriptive statistics, continuous variables were reported as median value with interquartile range or means with standard deviation [SD]. Categorical variables were instead expressed as number of events and percentage, dividing the number of events by the total number excluding missing and unknown cases. Differences between the two groups were explored using the Mann-Whitney U-test or Student *t* test for continuous variables. Differences between proportions were assessed by Fisher’s exact test or the χ^2^ test, when appropriate.

A NADE nomogram model was generated to predict the probability of 6-month unfavorable outcome. To develop a nomogram, the pre-established predictors and all variables with a probability value < 0.20 in the univariate analysis were entered into a multivariate logistic regression model. A final model selection was carried out by a backward stepdown selection process with the Akaike information criterion. Regression coefficients and odds ratios (OR) with two-sided 95% confidence intervals (95% CIs) for each of the variable included in the model were finally calculated. Collinearity of combinations of variables that entered the multivariate logistic regression analysis was assessed by the Condition Index (< 30 being considered non-significant) and Variation Inflation Factors (VIF, < 2 being considered non-significant). The performance of the model was assessed by discrimination (the ability of a proposed model to separate patients with different outcomes) and calibration (the relative distance of predictions from actual outcome). The predictive accuracy of the nomogram model was assessed by calculation of the area under curve (AUC) of the receiver-operating characteristic (ROC). Calibration was carried out using a calibration plot, in which the predicted probabilities were plotted against the frequency of the observed unfavorable outcome. The prediction of a well-calibrated model should be mirrored by a 45° diagonal line. Given that all predictive equations tend to be over-fitting to the original sample, the model was internally validated using bootstrap resampling. The statistical analysis was carried out using SPSS version 22.0 (IBM Corporation, Armonk, NY, USA), Stata version 13.0 (Stata Corporation, College Station, TX, USA) statistical software and the statistical software package R, version 3.3.3 (R Development Core Team, Auckland, New Zealand). All tests were two sided and *P* < 0.05 was considered statistically significant.

## Results

Based on the retrospective stroke registry, a single-center study from May, 2013 to May, 2018 was conducted in Nanjing First Hospital (China) for ischemic stroke within 12 h of symptoms onset. The final population consisted of 499 patients for acute ischemic stroke throughout the study period (median age 69 years; IQR 60–78 years). Unfavorable outcome (mRS score 3–6) after 6-month from acute ischemic stroke was observed in 140 (28.1%) patients, and within the follow-up period no patients died (mRS score = 6). The clinical, demographic and laboratory characteristics of the patients in the favorable outcome cohorts (*n* = 359) and unfavorable outcome (*n* = 140) cohorts are shown in Table 1 and Additional file 1: Table S1. In addition to age (65 versus 77; *p* < 0.0001), NIHSS score on admission (2 versus 5; *p* < 0.0001), previous diabetes mellitus (89 versus 54; *p* = 0.002) and creatinine (71 versus 78; *p* = 0.003), other factors such as gender, coronary artery disease, atrial fibrillation, previous stroke, previous antiplatelet, smoking, drinking, diastolic blood pressure (BP), international normalized ratios (INR), fasting blood glucose (FBG), triglyceride (TG), high density lipoprotein (HDL), and glycated hemoglobin (HbA1c) were also associated with unfavorable outcome in the univariate analysis. All variables with a probability value < 0.20 in the univariate analysis and traditional stroke risk factors such as previous hypertension, systolic BP and Body Mass Index (BMI) were added into the multivariate regression model.

**Table 1:** Demographics and clinical characteristics according to 6-month outcome

	Favorable outcome (mRS 0–2))	Unfavorable outcome (mRS 3–6)	*P*
Patients, n(%)	359	140	
Sex, n(%)			0.021^*^
Male, n(%)	254 (70.8)	84 (60.0)	
Female, n(%)	105 (29.2)	56 (40.0)	
Medical history, n (%)			
Hypertension	249 (69.4)	105 (75.0)	0.212^*^
Diabetes mellitus	89 (24.8)	54 (38.6)	0.002^*^
Hyperlipidemia	8 (2.2)	5 (3.6)	0.397
Coronary artery disease	38 (10.6)	33 (23.6)	< 0.0001^*^
Atrial fibrillation	23 (6.4)	24 (17.1)	< 0.0001^*^
Previous stroke	68 (18.9)	42 (30.0)	0.007^*^
Baseline data			
Age (years), median (IQR)	65 (58–74)	77 (70–83)	< 0.0001^*a^
NIHSS score, median (IQR)	2 (1–4)	5 (2–10)	< 0.0001^*a^
BMI, kg/m^2^, mean (SD)	24.79 (3.25)	24.51 (3.76)	0.407^*^
Systolic BP, mmHg, median (IQR)	140 (130–160)	140 (130–158)	0.684^*a^
Creatinine, umol/L, median (IQR)	71 (60–84)	78 (63.25–95.25)	0.003^*a^

*mRS* modified Rankin Scale, *NIHSS* National Institute of Health stroke scale, *BMI* Body Mass Index

^*^included into the multiple logistic regression models (*P* < 0.2) and Additionally traditional stroke risk factors such as Hypertension, Systolic BP and BMI were added into the model.

^a^Calculated using Mann-Whitney U test. BMI Calculated using Student’s t tests

**Fig. 1:**
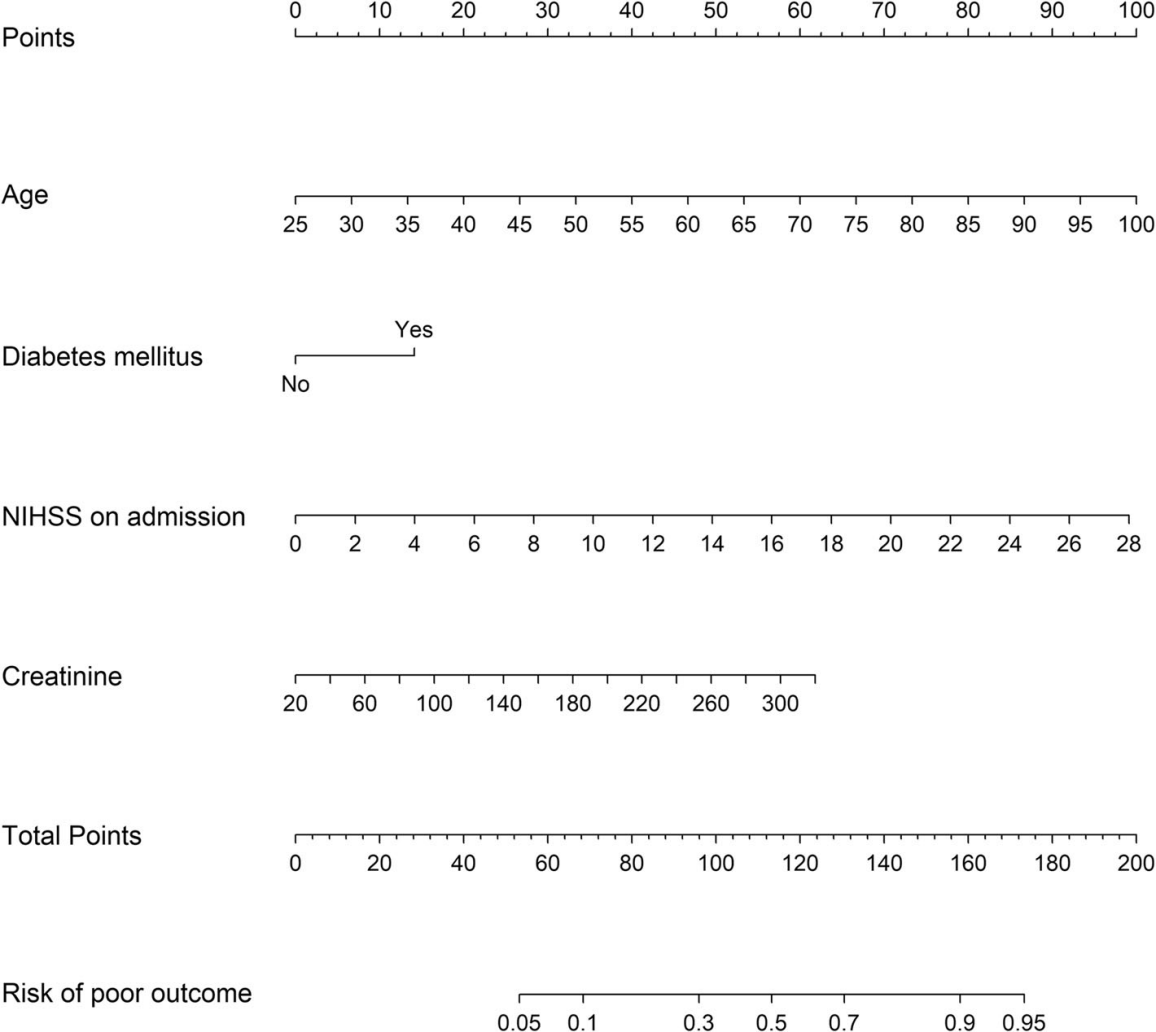
The NADE nomogram for predicting 6-month unfavorable outcome following ischemic stroke in Chinese patients. NIHSS: National Institutes of Health Stroke Scale

In multivariate analysis, NIHSS score on admission (OR: 1.190, 95%CI: 1.125–1.258), age (OR: 1.068, 95%CI: 1.045–1.090), previous diabetes mellitus (OR: 1.995, 95%CI: 1.236–3.221) and creatinine (OR: 1.010, 95%CI: 1.002–1.018) were entered into a logistic regression model to construct the NADE nomogram for prediction of the probability of 6-month poor outcome in the cohort after the acute ischemic event (Table 2, Fig. 1). No significant statistical collinearity was observed for any of the four independent risk factors that entered the multivariate logistic regression analysis. The logistic regression model resulted: Log [p(x)/1-p(x)] = − 7.388 + (0.174 × NIHSS score) + (0.065 × age) + (0.691× previous diabetes mellitus) + (0.010 × creatinine); where p(x) was the probability of 6-month unfavorable outcome.

**Table 2:** significant predictors of 6-month unfavorable outcome after an acute ischemic stroke

	OR	Error	Wald	*P*	95% CI
Age	1.068	0.012	6.07	< 0.0001	1.045–1.090
NIHSS on admission	1.190	0.034	6.07	< 0.0001	1.125–1.258
Creatinine	1.010	0.004	2.46	0.014	1.002–1.018
Previous Diabetes mellitus	1.995	0.488	2.83	0.005	1.236–3.221

**Fig. 2:**
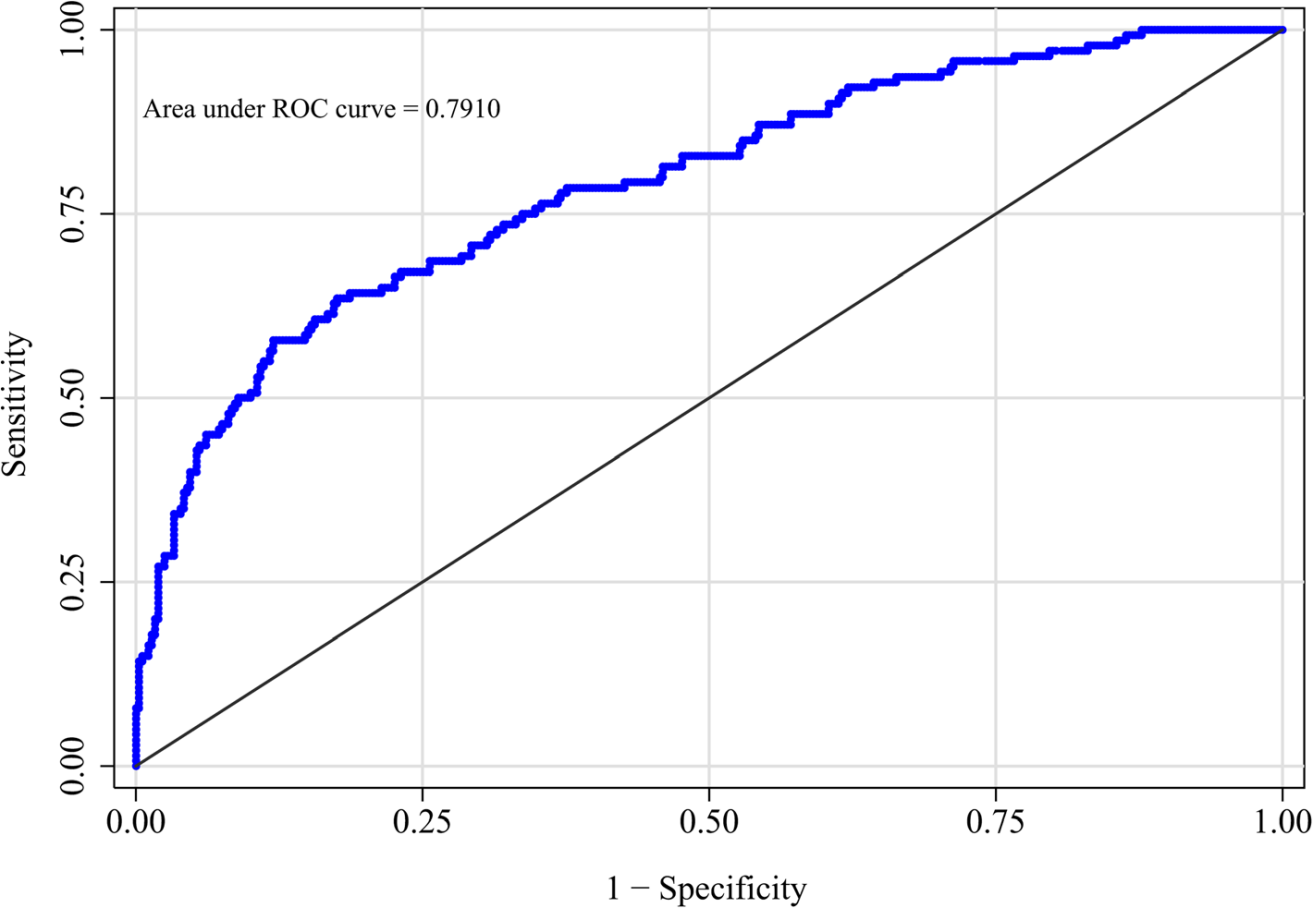
Receiver operating characteristic (ROC) curve of the nomogram for predicting 6-month unfavorable outcome following ischemic stroke in Chinese patients

The nomogram was developed by assigning a graphic initial score to each of the four independent prognostic factors with a point range from 0 to 100, which was then summed to create a total score, finally converted into an individual risk of 6-month unfavorable outcome expressed in percentage, thus ranging from 0 to 100%. It was predicted that the higher total score of the nomogram was associated with the higher likelihood of unfavorable outcome, while the lower total score was associated with the lower likelihood of adverse outcome. The AUC-ROC value of the NADE nomogram was 0.791 (95% CI: 0.746–0.836) in the cohort (Fig. 2). The age values and NIHSS scores on admission exhibited a good diagnostic accuracy for identifying patients with unfavorable outcome after 6 months, displaying an AUC of 0.734 (95% CI 0.683–0.785; *p* < 0.0001) and 0.713 (95% CI 0.662–0.764; *p* < 0.0001), respectively.

The total number of patients with a risk probability < 10% was 116/499 (23.2%), and only 9 of these had an unfavorable outcome (0.936 sensitivity, 0.298 specificity, 0.922 negative predictive value and 0.342 positive predictive value). The total number of patients with a risk probability < 40% was 386/499 (77.4%), 66 of whom had unfavorable outcome (0.529 sensitivity, 0.891 specificity, 0.829 negative predictive value and 0.655 positive predictive value). Finally, the total number of patients with a high-risk probability (i.e., > 80%) was 25/499 (5%), the vast majority of whom (21/25; 84%) had a poor prognosis (0.15 sensitivity, 0.989 specificity, 0.749 negative predictive value and 0.84 positive predictive value).

**Fig. 3:**
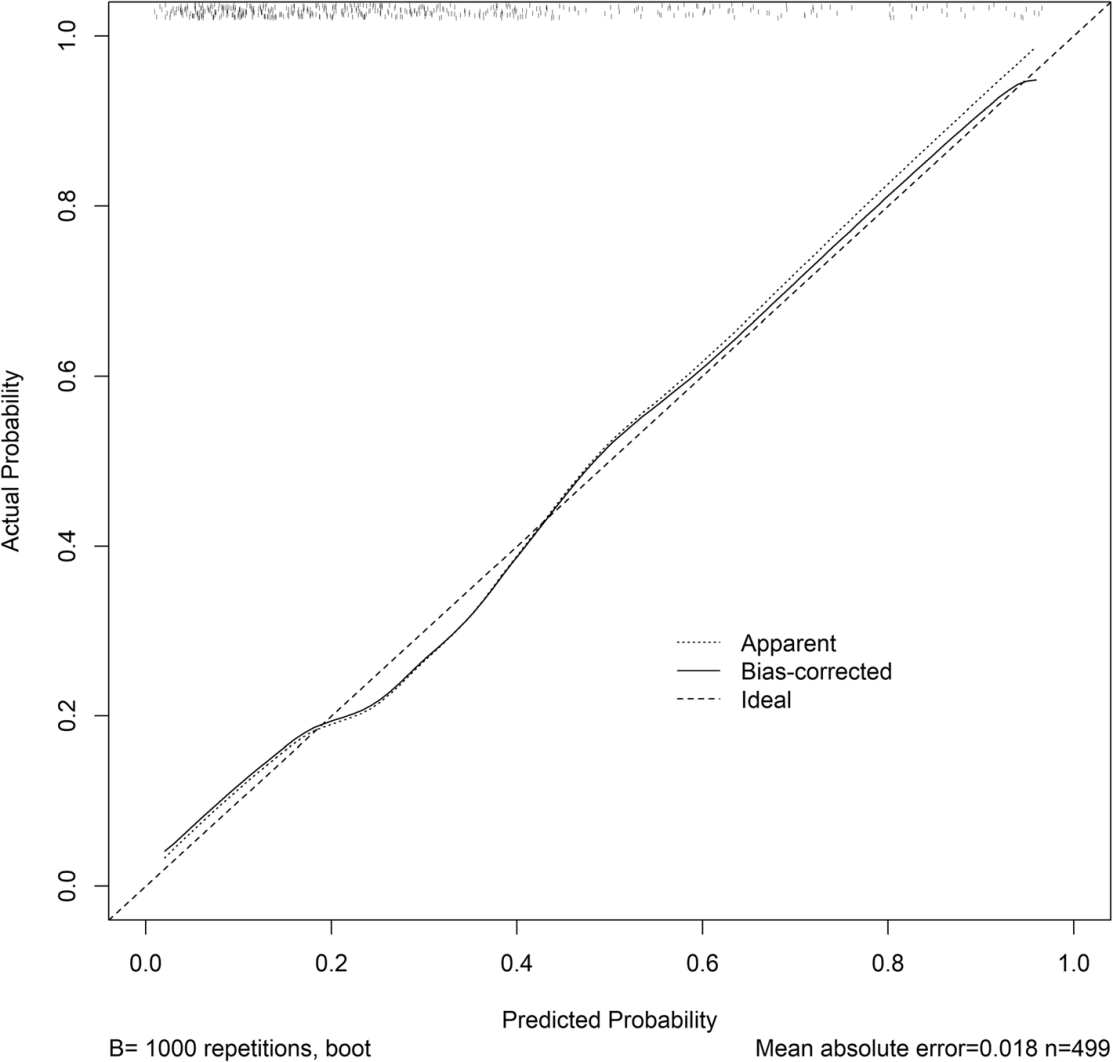
The calibration plot for the nomogram for predicting 6-month unfavorable outcome following ischemic stroke in Chinese patients. Dashed line is reference line where an ideal nomogram would lie. Dotted line is the performance of nomogram, while the solid line corrects for any bias in nomogram

The model was internally validated using 1000 bootstrap samples to calculate the discrimination with accuracy, and the good predictive performance of the nomogram was confirmed, yielding a notable AUC of 0.791 (95% CI 0.744–0.837; *p* < 0.0001) (Fig. 2). The bias-corrected calibration plot for the nomogram model showed the adequate agreement between predictors calculated with the NADE nomogram and actual unfavorable outcomes at the end of the follow-up period (Fig. 3). Calibration graphic revealed adequate fit of the model predicting the risk of poor prognosis at 6-month. The Hosmer-Lemeshow goodness-of-fit test showed good calibration of the nomogram (*p* = 0.4982).

## Discussion

Ischemic stroke is one of the leading causes of mortality and disability, and post-stroke disability has now become a public health care problem, so that the early and reliable identification of unfavorable outcome after ischemic stroke should be a valuable perspective on precise clinical and therapeutic management.

Some earlier models and prognostic scores identified that NIHSS score [12–16], age [12–14, 16, 17], creatinine [18, 19] and diabetes mellitus [14, 16, 20], size infarct [8] were independent predictor of unfavorable outcome in stroke patients. However, these above models and scores were limited by the use of dichotomization / categorization of predictors, because the process of categorizing discrete/continuous variables (size infarct, age and NIHSS score) into 2 or 4 risk groups was often statistically inefficient and may decrease the predictive accuracy. Moreover, the disadvantage of dichotomization is that it does not make use of within-category information and lead to the loss of information.

Using continuous variables, we are the first to present a visual NADE nomogram which is a better tool for individualized prediction of the probability from 5 to 95% of 6-month poor outcome following ischemic stroke in Chinese patients, as shown in Fig. 1. The NADE nomogram, defined as graphical computation instrument composing four predictors that age, NIHSS score on admission, creatinine, and diabetes mellitus were observed to be independently associated with 6-month unfavorable outcome for individual ischemic stroke, and the effect remained significant with good discriminative performance even after adjusting other clinical, and demographic variables. That is, we found that NIHSS score on admission, age, previous diabetes mellitus and creatinine were significant and independent predictors of poor outcome. Moreover, patients age, NIHSS score on admission and creatinine are the non-categorical variables by order of decreasing multivariate predictive effect on the probability of 6-month poor outcome with good discriminative performance for individual stroke patients.

The NADE nomogram was a reliable model to evaluate the risk of 6-month mRS score 3–6 in stroke individuals, which showed a significant superiority for prediction of unfavorable outcome. Differently from prognostic scores, The NADE nomogram assigns a probability (from 5 to 95%) of unfavorable outcome. For example, the NADE nomogram assigned > 95% probability of adverse consequence in an 80-year-old patient (74 points) stroke patient, with a history of diabetes mellitus (14 points), NHISS score of 10 (35 points) and creatinine level of 300 (58 points), with a total score of 181 points. On the other hand, < 10% probability of unfavorable outcome was nominated to a 40 years old (20 points), with no history of diabetes mellitus (0 points), NHISS score of 5 (18 points) and creatinine level of 100 (17 point), with a total score of 55 points. By converting the total score into a continuum of individual probability, the NADE nomogram can reclassify the risk of poor outcome more precisely. So the NADE nomogram could be better than previous prognostic scores and models based on outdated risk-grouping categorization for identifying different risk predictors in patients. Thus the NADE nomogram may provide more detailed information to facilitate the early identification of patients with very high probability of poor outcome and to discuss prognosis with patients and their families.

The NADE nomogram integrated patient age, NIHSS score, creatinine and diabetes mellitus for individualized prediction of 6-month unfavorable outcome following ischemic stroke in Chinese patients. Firstly, we found that age and NHISS on admission are the two strongest and independent predictors. For example, the NADE nomogram assigns a 45% probability of unfavorable outcome to a patient over 70 years old with modest creatinine and diabetes mellitus whose NIHSS score is 6, respectively. Instead, 80-year-old (74 points) and NHISS score of 10 (35 points) substantially influence the outcome in the patient because the probability of unfavorable outcome may remain higher than 75%. Moreover, age contributed to a long-term mortality and poor outcome because old age is associated with some cardiovascular diseases such as coronary artery disease, high blood pressure and high cholesterol. In patients > 80 years of age, the risk of poor outcome could be increased by the increased prevalence of cerebral small-vessel disease and comorbidities. Indeed, the NIHSS score indicates a more severe stroke, which can ultimately lead to a poorer outcome, whereas age means a less intense recovery. Secondly, the NADES nomogram also found that diabetes mellitus in ischemic stroke could predict worse outcome independently, because diabetes mellitus can affect the blood vessels and the nerves, which increases the chance of developing poor outcome. Finally, we observed that increased serum creatinine level was associated with unfavorable clinical outcome. Serum creatinine concentration is widely used as an index of renal function, and impaired renal function is associated with poor outcome after stroke [19].

Our study has some limitations. Firstly, it was an exploratory study in a single center and not a randomized controlled trial (RCT) designed to investigate the critical factors affecting 6-month poor outcome, and the clinical significance of this study may be attenuated by its design. Secondly, data of known neurobiological predictors such as infarct size [8] were not available in the present study, which may affect the development of the NADE nomogram model to predict the probability of 6-month poor outcome. So the neuroimaging predictors should also be integrated into our model in future studies, which may increase its discriminative performance of the nomogram model. Finally, an external validation in a completely different cohort of patients is warranted although the discriminative performance of the NADE model was good. Despite these limitations, as far as we know, the present study is the first attempt to construct and validate a nomogram to predict the probability of 6-month poor outcome in Chinese stroke patients.

## Conclusions

The NADE nomogram may is a reliable tool with accurate continuous probability power to provide an individual and precise prediction of the risk probability of 6-month unfavorable outcome following ischemic stroke in Chinese patients.

## Supplementary information

**Additional file 1: Table S1.** Demographics and clinical characteristics according to 6-month outcome.

## Data Availability

The data sets in this study are available from the corresponding author on reasonable request.

## Abbreviations

AUC: The area under the curve
BMI: Body Mass Index
BP: Blood pressure
CI: Confidence interval
CT: Computed tomography
FBG: Fasting blood glucose
HbA1c: Glycated hemoglobin
HDL: High density lipoprotein
ICH: Intracranial hemorrhage
IQR: International normalized ratios
mRS: Modified Rankin Scale
NADE: **N**IHSS score on admission, **A**ge, previous **D**iabetes mellitus and cr**E**atinine
NIHSS: National Institute of Health stroke scale
OR: Odds ratios
RCT: Randomized controlled trial
ROC: Receiver-operating characteristic
SD: Standard deviation
TG: Triglyceride
VIF: Variation Inflation Factors
