# Supplementary material for: A NADE nomogram to predict the probability of 6-month unfavorable outcome in Chinese patients with ischemic stroke
Source: BMC Neurol. 2019 Nov 7;19:274. doi: 10.1186/s12883-019-1464-6 (PMC6839074; doi:10.1186/s12883-019-1464-6)
Supplement: Supplementary file 1 — Additional file 1: Table S1. Demographics and clinical characteristics according to 6-month outcome. [file 12883_2019_1464_MOESM1_ESM.docx]

| **Additional file 1** Demographics and clinical characteristics according to 6-month outcome | | | |
| --- | --- | --- | --- |
|  | Favorable outcome  (mRS 0–2)) | Unfavorable outcome  (mRS 3–6) | *P* |
| Patients, n(%) | 359 | 140 |  |
| IV thrombolysis, n(%) |  |  | 0.836 |
| No thrombolysis | 279(77.7) | 110(78.6) |  |
| Thrombolysis | 80(22.3) | 30(21.4) |  |
| Interval from onset to hospital within 4.5h, n(%) | 86(24.0) | 35(25.0) | 0.807 |
| Previous antiplatelet, n(%) |  |  | 0.088* |
| No antiplatelet | 318(88.6) | 116(82.9) |  |
| antiplatelet | 41(11.4) | 24(17.1) |  |
| Smoking, n (%) |  |  | 0.005* |
| Never smoker | 156(43.5) | 77(55.0) |  |
| Former smoker | 54(15.0) | 27(19.3) |  |
| Current smoker | 149(41.5) | 36(25.7) |  |
| Drinking, n (%) |  |  | 0.001* |
| Never drinker | 201(56.0) | 97(69.3) |  |
| Former drinker | 40(11.1) | 20(14.3) |  |
| Current drinker | 118(32.9) | 23(16.4) |  |
| Baseline data |  |  |  |
| Diastolic BP, mmHg, median (IQR) | 85(80-97) | 80(80-90) | 0.044*# |
| Platelet count, 10^9^/L, median (IQR) | 195(165-232) | 193.5(160.25-240.5) | 0.701# |
| INR, median (IQR) | 0.95(0.90-1.00) | 0.985(0.95-1.04) | <0.0001*# |
| FBG, mmol/L, median (IQR) | 5.18(4.64-6.37) | 5.42(4.655-7.20) | 0.077*# |
| TC, mmol/L, median (IQR) | 4.42(3.69-5.10) | 4.205(3.705-4.983) | 0.250# |
| TG, mmol/L, median (IQR) | 1.36(0.95-2.04) | 1.22(0.883-1.735) | 0.018*# |
| HDL, mmol/L, median (IQR) | 1.05(0.91-1.21) | 1.00(0.84-1.198) | 0.089*# |
| LDL, mmol/l, median (IQR) | 2.75(2.08-3.31) | 2.64(2.11-3.23) | 0.594# |
| HbA1c, %, median (IQR) | 5.90(5.50-6.70) | 6.00(5.60-7.10) | 0.128*# |

mRS modiied Rankin Scale, BP blood pressure, INR International normalized ratios, FBG Fasting blood glucose, TC total cholesterol*,* TG triglyceride, HDL High density lipoprotein, LDL Low density lipoprotein, HbAc1 Glycated hemoglobin. *included into the multiple logistic regression models (*P*<0.2). # Calculated using Mann-Whitney U test.
